# High but slightly declining COVID-19 vaccine acceptance and reasons for vaccine acceptance, Finland April to December 2020

**DOI:** 10.1017/S0950268821001114

**Published:** 2021-05-11

**Authors:** Charlotte C. Hammer, Veronica Cristea, Timothee Dub, Jonas Sivelä

**Affiliations:** 1 Department of Health Security, Finnish Institute for Health and Welfare (THL), Helsinki, Finland; 2 European Programme for Intervention Epidemiology Training (EPIET), European Centre for Disease Prevention and Control (ECDC), Stockholm, Sweden

**Keywords:** COVID-19, KAP study, vaccine acceptance, vaccine hesitancy

## Abstract

We investigated likelihood to vaccinate and reasons for and against accepting a coronavirus disease 2019 (COVID-19) vaccine among adult residents of Finland. Vaccine acceptance declined from 70% in April to 64% in December 2020. Complacency and worry about side effects were main reasons against vaccination while concern about severe disease was a strong motive for vaccination. Convenience of vaccination and recommendations by healthcare workers were identified as enablers for vaccination among those aged under 50 years. Understanding barriers and enablers behind vaccine acceptance is decisive in ensuring a successful implementation of COVID-19 vaccination programmes, which will be key to ending the pandemic.

## Introduction

Vaccination will be a cornerstone together with non-pharmaceutical interventions to end the coronavirus disease 2019 (COVID-19) pandemic. However, vaccine hesitancy has the potential to hamper this effort. Vaccine hesitancy has been defined by the World Health Organization as ‘a delay in acceptance or refusal of vaccination despite availability of vaccination services’ [1].

Finland has started vaccination against COVID-19 in late December 2020. In order to facilitate the rollout, it is paramount to understand the population's knowledge, attitudes and perceptions (KAP) of the vaccination programme. Finland has conducted repeated KAP surveys throughout the pandemic using the WHO Office for Europe COVID-19 Snapshot Monitoring (COSMO) protocol [2]. These have also included questions on vaccination. Using these data, we assessed the likelihood of the population to accept a COVID-19 vaccine at four time points between April and December 2020 and investigated reasons for accepting the vaccine in November/December 2020. In Europe, similar studies, also under the umbrella of the COSMO, are currently conducted in Denmark [3, 4] and Germany [5, 6].

## Methods

We conducted four rounds of online surveys with approximately 1000 individuals each. The survey was pre-tested internally. Recruitment was separate for each round and recruitment and facilitation of the online survey was done by the Finnish polling company Taloustutkimus Oy. Data collection for round one was 7–9 April 2020 (*n* = 1009), for round two 24–28 April 2020 (*n* = 1032), for round three 08–11 May 2020 (*n* = 1060) and for round four 27 November–01 December 2020 (*n* = 1050). The respondents were representative of the Finnish 18–79-year-old population with regards to gender, age and geographic area based on latest population statistics [7]. Samples were formed by using random sampling for the requested target group within three commercial panels. To account for differences between different age groups in willingness to participate to surveys, and ensure representativeness, we sent relatively more invitations to younger panel members and used quota (gender, age and geographic area) sampling. Further information about the main commercial panel used can be found in the supplementary material. The surveys covered questions regarding behaviours, perceptions, affect, knowledge and opinions, including a question on COVID-19 vaccine acceptance should one become available (‘If a vaccine for COVID-19 becomes available and is recommended for you, would you accept it?’). For the fourth round of the survey, we added questions regarding vaccine perceptions in general and factors that might influence the decision to accept a COVID-19 vaccine as these became more pertinent when vaccination shifted from a hypothetical to a realistic (in the very near future) scenario. Answers to the questions were on a 7-point Likert scale ranging from 1 (strong disagreement) to 7 (strong agreement). We performed descriptive statistical analysis of the individual questions, stratified by age and gender. We also did a multiple linear regression analysis of the likelihood to accept a COVID-19 vaccine and potential predictors. We calculated estimates and 95% confidence intervals and assumed statistical significance at *P* < 0.05. Analysis was done in R (version 3.6.1) using RStudio (version 1.2.5001).

## Results

**Fig. 1.**
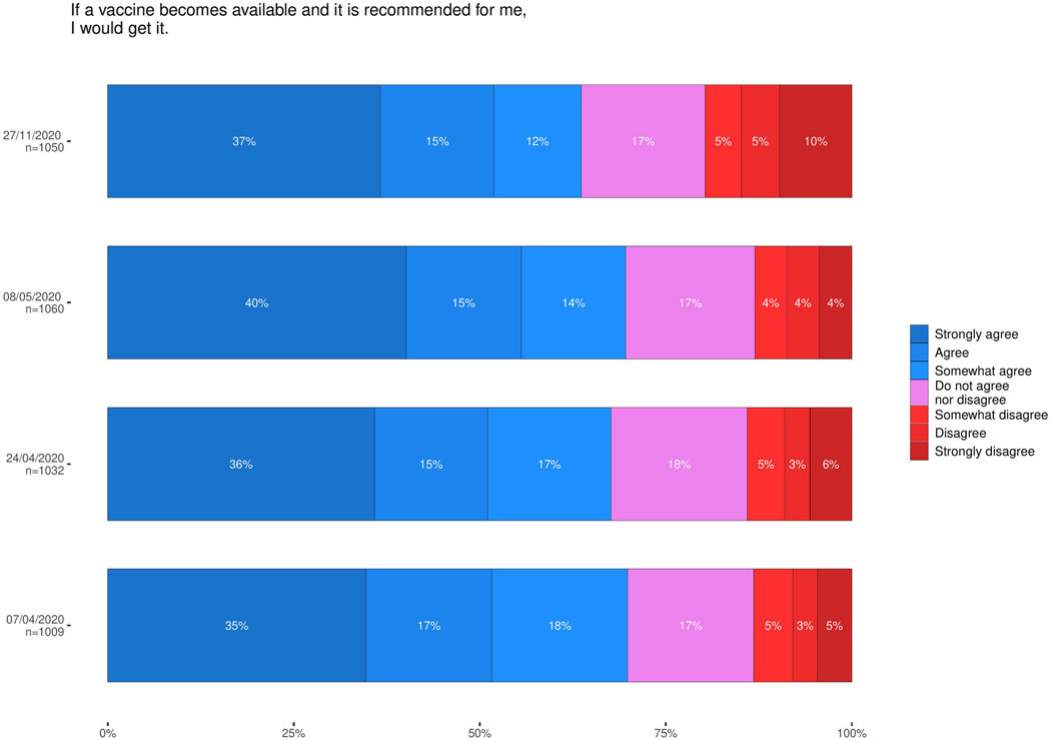
Self-declared likelihood of accepting a COVID vaccine if/when one is offered for all four rounds of the survey.

The percentage of persons strongly agreeing to accept a COVID-19 vaccine should one be offered to them has not changed much between April and December with 35% responding thus in early April and 37% in November/December. However, the percentage of persons strongly disagreeing with accepting a vaccine should one be offered to them has doubled, from 5% in early April to 10% in November/December (4% in late April and 6% in May) (Fig. 1).

**Fig. 2.**
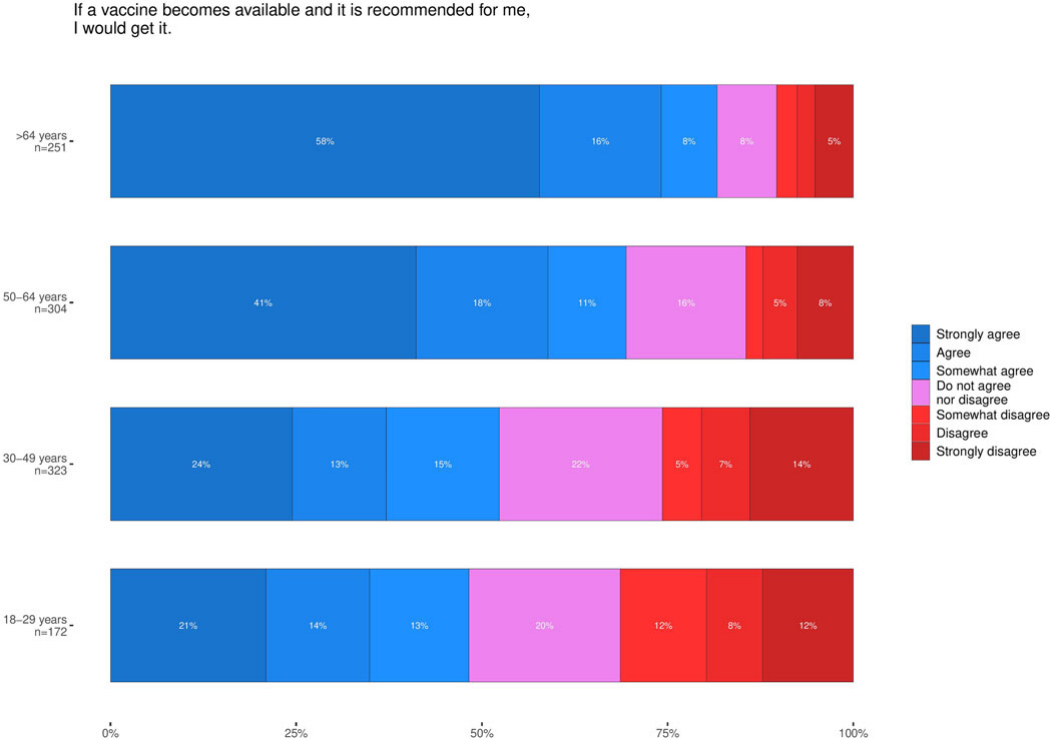
Self-declared likelihood of accepting a COVID vaccine if/when one is offered during the fourth round (November/December 2020) by age group.

The percentage of persons agreeing to receive a COVID-19 vaccine if/when one would be recommended declined from 70% (95% CI 67–73%) in April to 64% (61–67%) in December, while the percentage of respondents disagreeing to receive it increased from 13% (11–15%) to 20% (17–22%) (Fig. 1). In the most recent round, the likelihood to strongly agree with accepting a vaccine should one be offered had a clear age gradient from 21% agreement in the young adult group (18−29 years) to 58% in the oldest group (over 64 years) (Fig. 2).

**Fig. 3.**
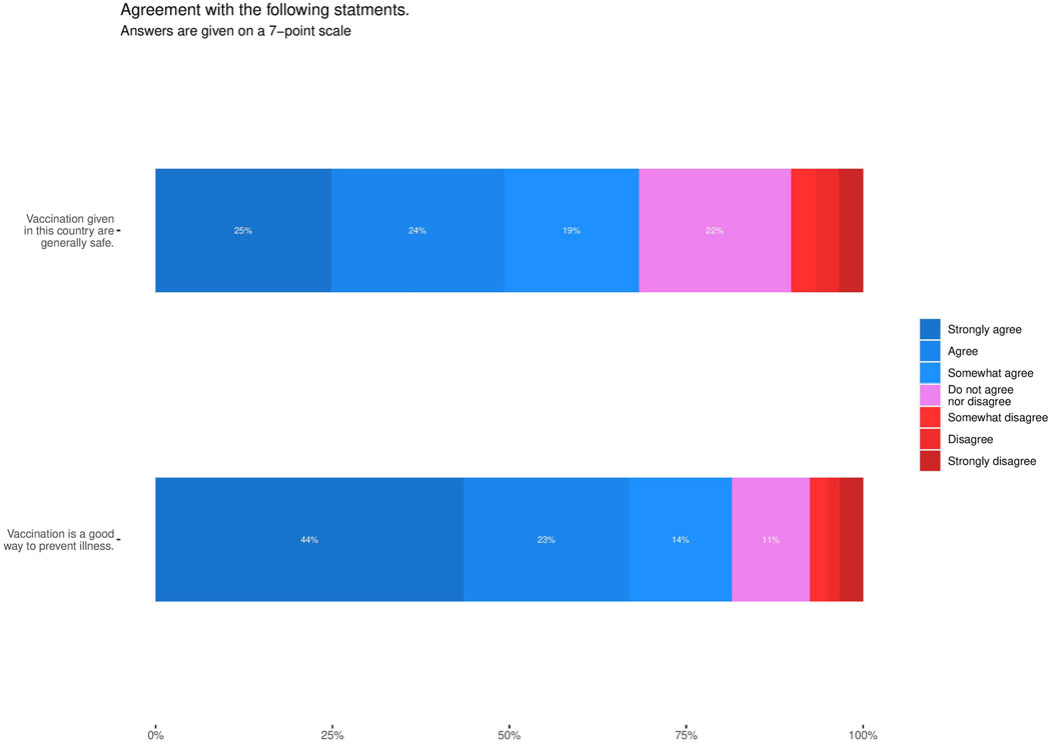
Agreement with general statements about vaccination during the fourth round (November/December 2020, *n* = 1050).

In November/December, we also inquired about more general perceptions regarding vaccination. Sixty-eight per cent of respondents agreed that vaccines administered in Finland are generally safe and 81% agreed that vaccination is a good way to prevent disease (Fig. 3). Among respondents not willing to receive the COVID-19 vaccine, 30% still agreed that vaccines administered in Finland were safe, while 44% agreed that vaccination is a good way to prevent diseases.

In order to better understand the possible motivation for accepting or rejecting an offered COVID-19 vaccine, we investigated the association of various predictors with the self-declared likelihood to accept a vaccine should one be offered (Table 1). Predictors included (dis)agreement to the two above-mentioned statements regarding vaccine safety and vaccination being a good way to prevent illness, demographic characteristic, factors that respondents took into consideration when making a decision about accepting the vaccine (see Table 1 for all included factors), as well as (dis)agreement with statements regarding conspiracy theories and public. We stratified the analysis depending on age (below 50 years and 50 years or older). We selected this cut-off as this was the point at which vaccine acceptance shifted from strongly in favour to more uncertain (see Fig. 2). In the above-50 age group, increased agreement that vaccines given in Finland are safe was associated with increased likelihood to vaccinate (estimate: 0.20, 95% CI 0.11–0.29, *P* < 0.001). This was, however, not the case in the younger age group, where we observed no significant association. Agreement that vaccination is a good way to prevent disease was significantly associated with increased likelihood to get vaccinated in both age strata (estimate: 0.38, 95% CI 0.25–0.52 below 50; estimate: 0.55, 95% CI 0.44–0.65 50 and above; *P* < 0.001 for both). If a person in the younger stratum considered the infection situation in Finland, which was better than most European countries with a weekly incidence per 100 000 of 54.71 [8], they were significantly less likely to accept the vaccine (estimate: −0.19; 95% CI −0.21 to −0.02; *P* = 0.016). This was, however, not observed in the older stratum and can be interpreted as complacency in the younger age group. For both groups the assumed protection of oneself was a reason to get vaccinated (estimate: 0.17, 95% CI 0.02–0.31, *P* = 0.022 below 50; estimate: 0.16, 95% CI 0.03–0.29, *P* = 0.016 50 and above). Similarly, worries about potential side effects reduced the likelihood in both strata (estimate: −0.27; 95% CI −0.35 to −0.19 below 50; estimate: −0.24, 95% CI −0.30 to −0.18 50 and above; *P* < 0.001 for both groups). Considering a recommendation from a healthcare worker was associated with an increased likelihood only in the younger stratum (estimate: 0.22, 95% CI 0.07–0.37, *P* = 0.005). This is particularly important as such a recommendation might therefore only have an impact in the <50-year-old group. Both groups were more likely to vaccinate if worried about severe disease (estimate: 0.11, 95% CI 0.03–0.19, *P* = 0.016 below 50; estimate 0.08, 95% CI 0.01–0.15, *P* = 0.021 50 and above). However, the ease of getting vaccinated was only significant in the younger stratum (estimate: 0.12, 95% CI 0.05–0.19, *P* = 0.001). We only observed an impact of gender in the older group, with women over the age of 50 being less likely to accept vaccination (estimate: −0.22, 95% CI −0.41 to −0.04, *P* = 0.019) compared to males.

**Table 1.** Association between self-declared likelihood to accept a COVID vaccine if/when one is offered (POL VACCINE) and predictors, stratified by age group

	Younger than 50 years old			Older than 50 years old		
	Likelihood to get vaccinated			Likelihood to get vaccinated		
Predictors	Estimates	CI95	*P*	Estimates	CI95	*P*
Agreement with: Vaccines are effective and safe	0.10	−0.23 to 0.23	0.108	0.20	0.11–0.29	<0.001***
Agreement with: Vaccination is a good way to protect against diseases	0.38	0.25–0.52	<0.001***	0.55	0.44–0.65	<0.001***
Agreement with importance for decision making: How well the vaccine protects against coronavirus disease	0.05	−0.08 to 0.18	0.415	0.01	−0.07 to 0.09	0.789
Agreement with importance for decision making: Infection situation in Finland	−0.12	−0.21 to −0.02	0.016**	−0.03	−0.10 to 0.05	0.479
Agreement with importance for decision making: The fact that the vaccine protects onself against coronavirus disease	0.17	0.02–0.31	0.022**	0.16	0.03–0.29	0.016**
Agreement with importance for decision making: The fact that the vaccine protects other people	0.10	−0.02 to 0.22	0.089	0.08	−0.02 to 0.19	0.104
Agreement with importance for decision making: Possible side effects of the vaccine	−0.27	−0.35 to −0.19	<0.001***	−0.24	−0.30 to −0.17	<0.001***
Agreement with importance for decision making: A recommendation from a healthcare professional	0.22	0.07–0.37	0.005**	0.02	−0.12 to 0.15	0.813
Agreement with importance for decision making: A recommendation from health authorities	0.12	−0.03 to 0.27	0.116	0.12	−0.01 to 0.25	0.071
Agreement with importance for decision making: Conversations with family and friends	−0.04	−0.12 to 0.05	0.395	−0.01	−0.08 to 0.05	0.733
Agreement with importance for decision making: How easy it is to get vaccinated	0.12	0.05–0.20	0.001**	0.05	−0.0 to 0.11	0.055
Own assessment of susceptibility of infection	−0.03	−0.14 to 0.09	0.646	−0.02	−0.10 to 0.06	0.688
Own assessment of probability of infection	0.03	−0.07 to 0.13	0.566	0.02	−0.06 to 0.10	0.601
Own assessment of severity if infected	0.10	0.02–0.19	0.016**	0.08	0.01–0.15	0.021**
Agreement with: Many very important things happen in the world that are never communicated to the public	−0.07	−0.16 to 0.02	0.141	−0.06	−0.13 to 0.00	0.053
Agreement with: Politicians usually do not tell us the true motives for their decisions	0.10	0.00–0.20	0.051	0.01	−0.06 to 0.08	0.712
Agreement with: The coronavirus is manmade and spread on purpose	0.04	−0.06 to 0.13	0.441	0.00	−0.07 to 0.08	0.903
Agreement with: Events which superficially seem to lack a connection are often the result of secret activities	−0.06	−0.16 to 0.03	0.198	0.05	−0.02 to 0.11	0.149
EDUCATION: primary school	Reference			Reference		
EDUCATION: vocational school	0.04	−0.46 to 0.54	0.865	−0.25	−0.60 to 0.10	0.162
EDUCATION: secondary school	0.23	−0.30 to 0.77	0.865	−0.05	−0.52 to 0.42	0.834
EDUCATION: college	0.27	−0.45 to 1.00	0.455	−0.13	−0.50 to 0.24	0.502
EUDCATION: polytech	−0.13	−0.65 to 0.39	0.625	−0.06	−0.47 to 0.35	0.791
EDUCTAION: university	0.27	−0.27 to 0.80	0.331	0.02	−0.35 to 0.38	0.913
GENDER: male	Reference			Reference		
GENDER: female	−0.21	−0.46 to 0.04	0.097	−0.22	−0.41 to −0.04	0.019**
GENDER: other	−0.90	−3.42 to 1.62	0.484	No	Observation	
AGEGROUP: 18–29 years old	Reference			No	Observations	
AGEGROUP: 30–39	−0.12	−0.42 to 0.18	0.438	No	Observations	
AGEGROUP: 40–49	0.05	−0.25 to 0.35	0.747	No	Observations	
AGEGROUP: 50–59 years old	No	Observations		Reference		
AGEGROUP: 60–69	No	Observations		0.10	−0.11 to 0.31	0.329
AGEGROUP: 70–79	No	Observations		−0.02	−0.27 to 0.23	0.855
Observations	474			547		
*R*^2^/*R*^2^ adjusted	0.631/0.612			0.691/0.678		

Non-demographic predictors and outcomes were on a scale from 1 to 7. Significance levels: ***P ⩽ 0.001; **P ⩽ 0.005.

## Discussion and conclusions

Over the course of our study, we have found a slight reduction in willingness to get vaccinated against COVID-19 among the Finnish population. Particularly the group stongly disagreeing to get vaccinated grew to twice its original size between April and December 2020. Additionally, we have found that the likelihood to agree to get vaccinated increases with age, with a particular change in the likelihood profile happening at between the age groups 40−50 years old and 50−64 years old.

A previous study from Finland has shown that trusting the safety of the potential vaccine is the strongest predictor of COVID-19 vaccination intention [9]. We did not specifically assess this predictor but that worries about potential side effects were a strong predictor of reduced likelihood to get vaccinated certainly aligns with it. Globally, 71.5% of people are at least somewhat likely to accept a COVID-19 vaccine should one be offered to them [10]. Finland in November/December 2020 was slightly below this with 64% of respondents agreeing at least somewhat. However, since the end of our study, these numbers seem to have risen and actual vaccine acceptance has been high, particularly among older groups, with more than 97% of over-85-year-olds in Helsinki and over 70% of over 80-year-olds Finland-wide having had their first dose already in March 2021 [11]. At the same time, just 49% of persons under the age of 30 said they would certainly get the vaccine in March [11]. Our findings regarding the role of side effects and worries of severe disease are highly expected and in line with the results from similar studies [9, 10]. Previous research has demonstrated that measures such as reducing barriers [12], or having conversations with trusted healthcare workers [13, 14] have been shown to be effective. However, both of these measures would in our case only increase vaccine uptake among the younger age group. That increased convenience and recommendations by healthcare workers would only work in the below 50 years group is potentially problematic as COVID-19 severity increases with age [8].

It is extremely important to understand the barriers and enablers for vaccine acceptance, particularly in a pandemic situation, as vaccinating the majority of the people is considered the key in ending the pandemic. This has impacts on logistical planning, communications strategies and to develop context-specific measures to overcome any barriers. It is therefore decisive in ensuring a successful implementation of vaccination programmes. As the reasons for accepting and refusing vaccination can be expected to change over time with new information regarding the vaccines becoming available and potential changes in the epidemiological situation, updates will become necessary. Therefore, it is essential to create mechanisms for monitoring these reasons rapidly and repeatedly during a pandemic.

### Limitations

This study is subject to some limitations. The obvious bias of an internet survey conducted by a polling company comes with the group of respondents who while representative of the Finnish population in terms of age, gender and location might not be representative in terms of views and opinions. Additionally, while being representative for regional diversity, the study was not powered for assessment of regional differences in opinions. However, the bigger limitation here lies within the topic which can only to a very limited degree be assessed quantitatively.

### Outlook

We have made positive experiences with the COSMO study design and in addition welcome that it will allow for cross-border comparison and collaboration across the WHO European region. This will be crucial in the coming months while we continue to monitor COVID-19 vaccine acceptance and hesitancy across Europe and globally. Finally, we strongly believe that additional qualitative studies are needed in order to better understand people's attitudes regarding vaccination and potential barriers and enablers for COVID-19 vaccination.

## Data Availability

Due to the very small number of respondents in some age and gender (other) categories, the data cannot be made publicly available as they are potentially identifying.
