# High but slightly declining COVID-19 vaccine acceptance and reasons for vaccine acceptance, Finland April to December 2020–Corrigendum

**DOI:** 10.1017/S0950268821001217

**Published:** 2021-06-04

**Authors:** Harlotte C. Hammer, Veronica Cristea, Timothee Dub, Jonas Sivelä

**Affiliations:** 1 Department of Health Security, Finnish Institute for Health and Welfare (THL), Helsinki, Finland; 2 European Programme for Intervention Epidemiology Training (EPIET), European Centre for Disease Prevention and Control (ECDC), Stockholm, Sweden

The original publication of this article was published with authors Timothee Dub and Jonas Sivelä assigned the incorrect affiliation. Their correct affiliation is affiliation 1, ^1^Department of Health Security, Finnish Institute for Health and Welfare (THL), Helsinki, Finland.

This has now been corrected in the original article.
